# Genesis and Symptom Interval of Osteosarcoma Following High-Energy Trauma

**DOI:** 10.7759/cureus.19068

**Published:** 2021-10-26

**Authors:** Mohd Ariff Sharifudin, Yam Chuan Khaw, Khairil Amir Sayuti, Wan Faisham Wan Ismail

**Affiliations:** 1 Orthopaedics and Traumatology, Faculty of Medicine, Universiti Sultan Zainal Abidin, Kuala Terengganu, MYS; 2 Orthopaedics, Hospital Universiti Sains Malaysia, Kota Bharu, MYS; 3 Radiology, Hospital Universiti Sains Malaysia, Kota Bharu, MYS; 4 Radiology, School of Medical Sciences, Kota Bharu, MYS; 5 Orthopaedics, Universiti Sains Malaysia, Kota Bharu, MYS

**Keywords:** osteosarcoma, high-energy trauma, symptom interval, genesis of sarcoma, bone sarcoma

## Abstract

We report three cases of femoral fracture, which subsequently developed osteosarcoma within the course of illness. The first patient presented with a spiral fracture treated with an interlocking femoral nail. He presented five months later with a painful knee swelling and was diagnosed with osteosarcoma. He was asymptomatic despite initial radiographs showed osteolysis at the distal metaphysis. The fracture united well and no evidence of marrow or soft tissue contamination. The second patient had a distal femoral fracture and underwent plate stabilization. Osteosarcoma developed at the united fracture site three years later. Both survived six to seven years without evidence of disease following the standard treatment protocol. The third patient had a closed distal third fracture treated with dynamic compression plating. He presented with an osteoblastic lesion in the proximal femur three years later. There was no initial radiological evidence of osteosarcoma one year before the clinical manifestation of the disease.

## Introduction

Symptom interval is defined as the time from the first onset of symptoms until a definite diagnosis is made and treatment is started [1,2]. The median symptom interval of osteosarcoma had been reported between one and 14.6 months [1,3,4]. Generally, osteosarcoma presents with painful progressive swelling of the affected region [4]. An asymptomatic incidental finding is yet to be reported.

We report three unique cases of osteosarcoma involving the femur following high-energy trauma. The first case presented with a fracture of the ipsilateral femur but was distant from the osteosarcoma lesion. The clinical manifestation appeared later after the fracture had completely healed. The second case presented with osteosarcoma, which developed later at the united fracture site. Another patient had osteosarcoma at the proximal femur during follow-up for ipsilateral distal femoral fracture. There was no clinical and radiological evidence one year before diagnosis. Our three cases are rare circumstances of osteosarcoma presentation and have broadened our knowledge about the genesis of this disease.

## Case presentation

Case 1

**Figure 1:**
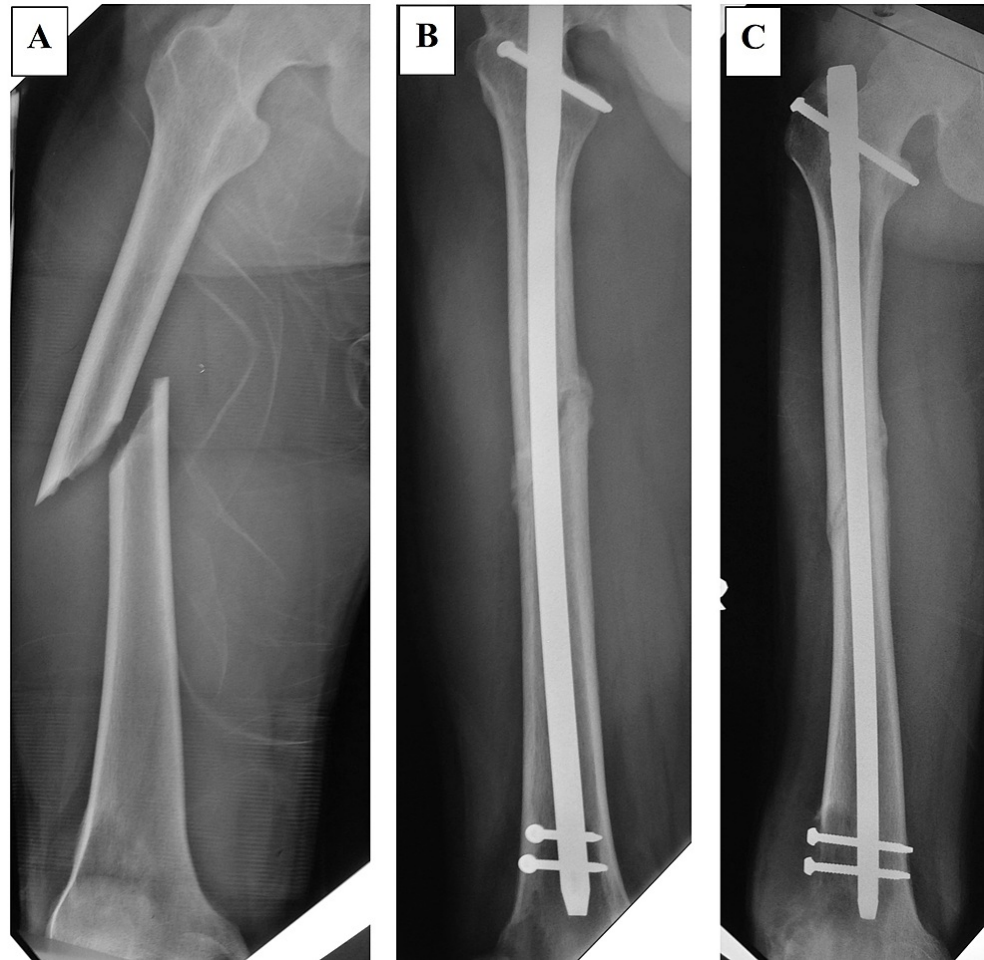
Pre-surgical and three-month post-interlocked nailing plain radiographs The patient sustained a traumatic spiral fracture of the midshaft of the right femur. There was no bony lesion or periosteal reaction seen at the fracture site (A). The fracture had united at three months after surgery with evidence of callus formation (B, C).

**Figure 2:**
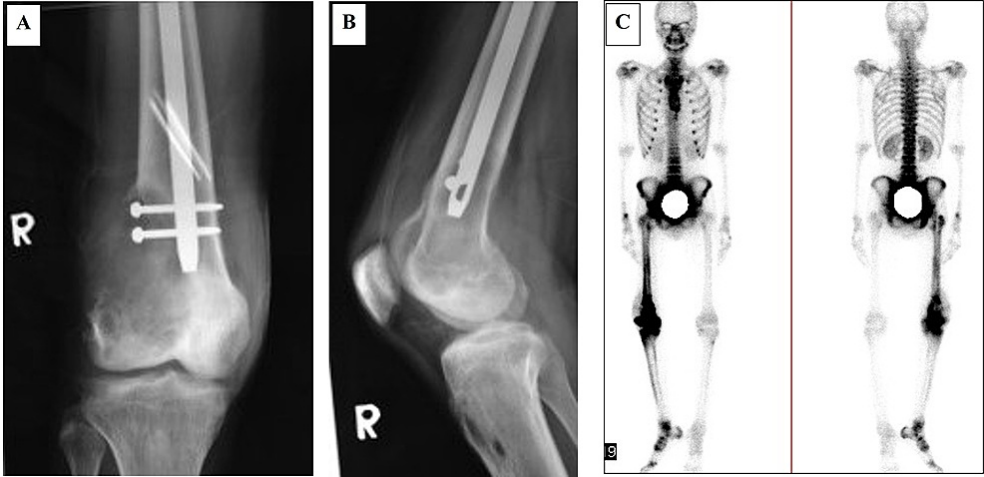
Evidence of an aggressive lesion at the distal right femur Cortical destruction involving the lateral aspect of the distal metaphysis extending into the epiphysis and extra-osseous component (A, B). Bone scan showed increased radiotracer uptake seen at the fracture site and the distal right femur. Increased radiotracer uptakes seen at the right hip and ankle joints, as well as the right foot, represent reactive hyperemic changes (C). R: right.

**Figure 3:**
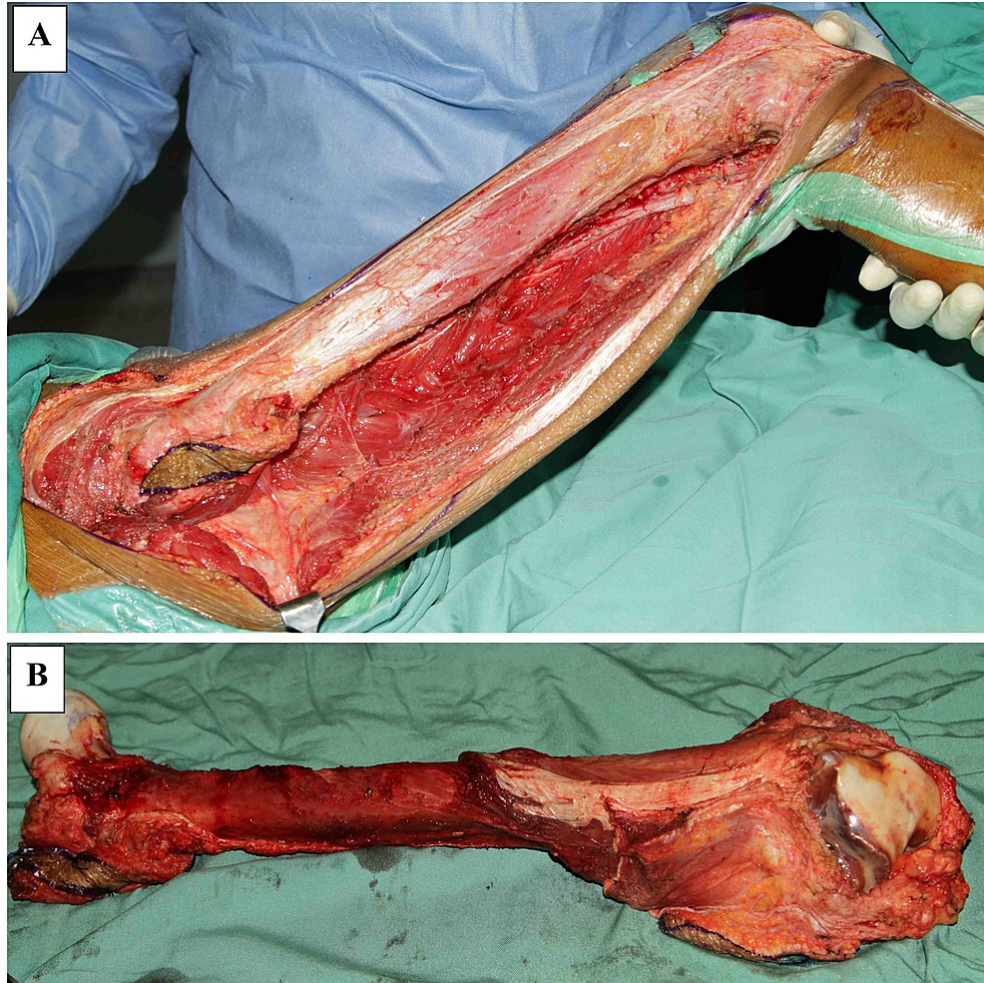
Intra-operative images of the first case during total femur resection The right femur before (A) and after total resection (B). No gross evidence of soft tissue infiltration or contamination at the fracture site and the proximal femur.

A 23-year-old gentleman presented with a spiral fracture of his right femur after being assaulted with a hammer (Figure 1A). He was treated with a femoral interlocking nail. Within 12 weeks, the fracture united and he regained full function of the limb (Figures 1B, 1C). Five months later, he had pain and swelling around his right knee. The plain radiograph showed a destructive lesion of the femoral metaphysis at the tip of the nail and the two locking screws (Figures 2A, 2B). Bone scan showed increased radiotracer uptake seen at the fracture site and the distal femoral meta-epiphysis region as well as its extra-osseous component (Figure 2C). Biopsy confirmed the diagnosis of osteosarcoma. There was no evidence of metastases on staging prior to commencement of chemotherapy, which was approximately eight months after the initial injury. He underwent three cycles of neoadjuvant chemotherapy and responded well. The neoadjuvant therapy used was according to the modified European Osteosarcoma Intergroup (EOI) Protocol consisting of a two-hour infusion of cisplatin 100 mg/m^2^ (total of three divided doses) administered on day one to day three and doxorubicin 25 mg/m^2^ administered over 24 hours via intravenous infusion on day one to day three. Total femur with distal quadriceps resection was performed to achieve a wide and adequate margin given previous surgery contamination. Intra-operatively, there was no evidence of soft tissue infiltration or contamination neither at the fracture site nor at the entry point for reaming and nail insertion (Figures 3A, 3B). The limb was reconstructed with total femur endoprosthesis and motorized latissimus dorsi flap. Post-surgery, the patient was subjected to both chemotherapy and radiotherapy. The remaining three cycles of the same chemotherapy regime as neoadjuvant were administered four weeks following the surgery. Local radiation of 60 centigray (cGy) was given to the entire femur to minimize local recurrence of oncological contamination from the initial interlocked nailing surgery.

**Figure 4:**
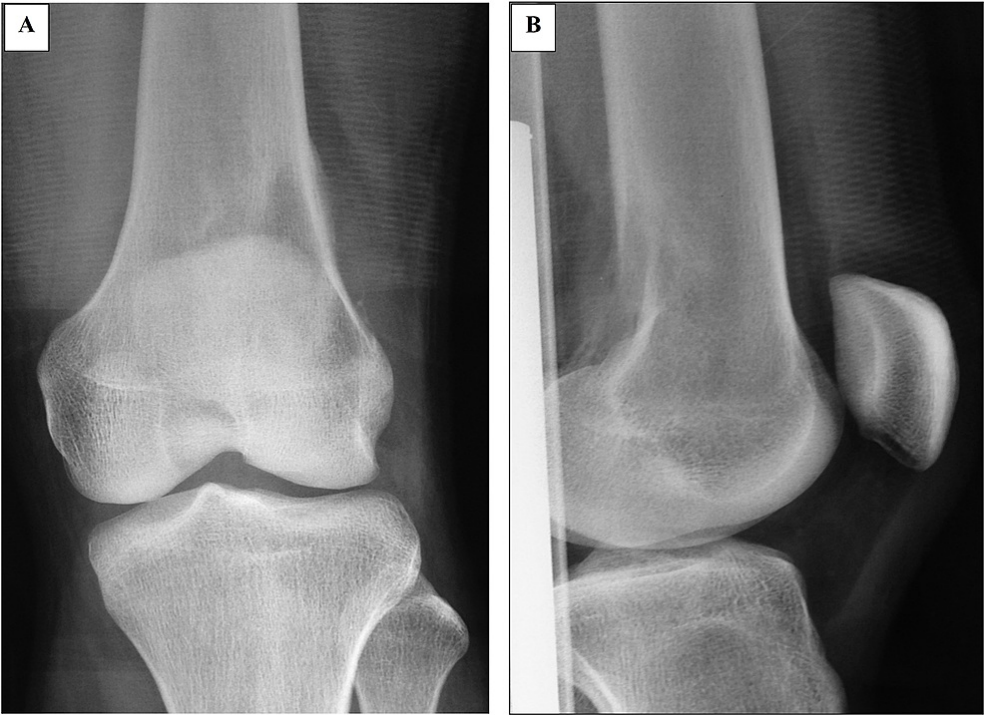
Plain radiographs of the right distal femur before the initial surgery Retrospective review of the anteroposterior (A) and lateral view (B) images revealed the presence of an ill-defined osteolytic lesion in the distal femoral metaphysis with minimal soft tissue opacity at its posterolateral aspect. The articular surface and space of the knee joint were preserved. These features were in keeping with an aggressive lesion involving the distal femur.

During his most recent follow-up, he was 60 months post-operation, free of disease, and ambulating without aid with a knee extension lag of 20 degrees due to the weakness of the quadriceps. Retrospective analysis of the initial knee radiograph following trauma revealed evidence of cancellous changes at the distal femoral metaphyseal region (Figures 4A, 4B). However, the patient did not complain of any symptoms prior to the injury he sustained. The fracture united well, and there was no evidence of bony destruction within the medullary cavity despite contamination from the previous cancerous area. Final histopathology confirmed that there was no evidence of marrow and proximal soft tissue infiltration. 

Case 2

**Figure 5:**
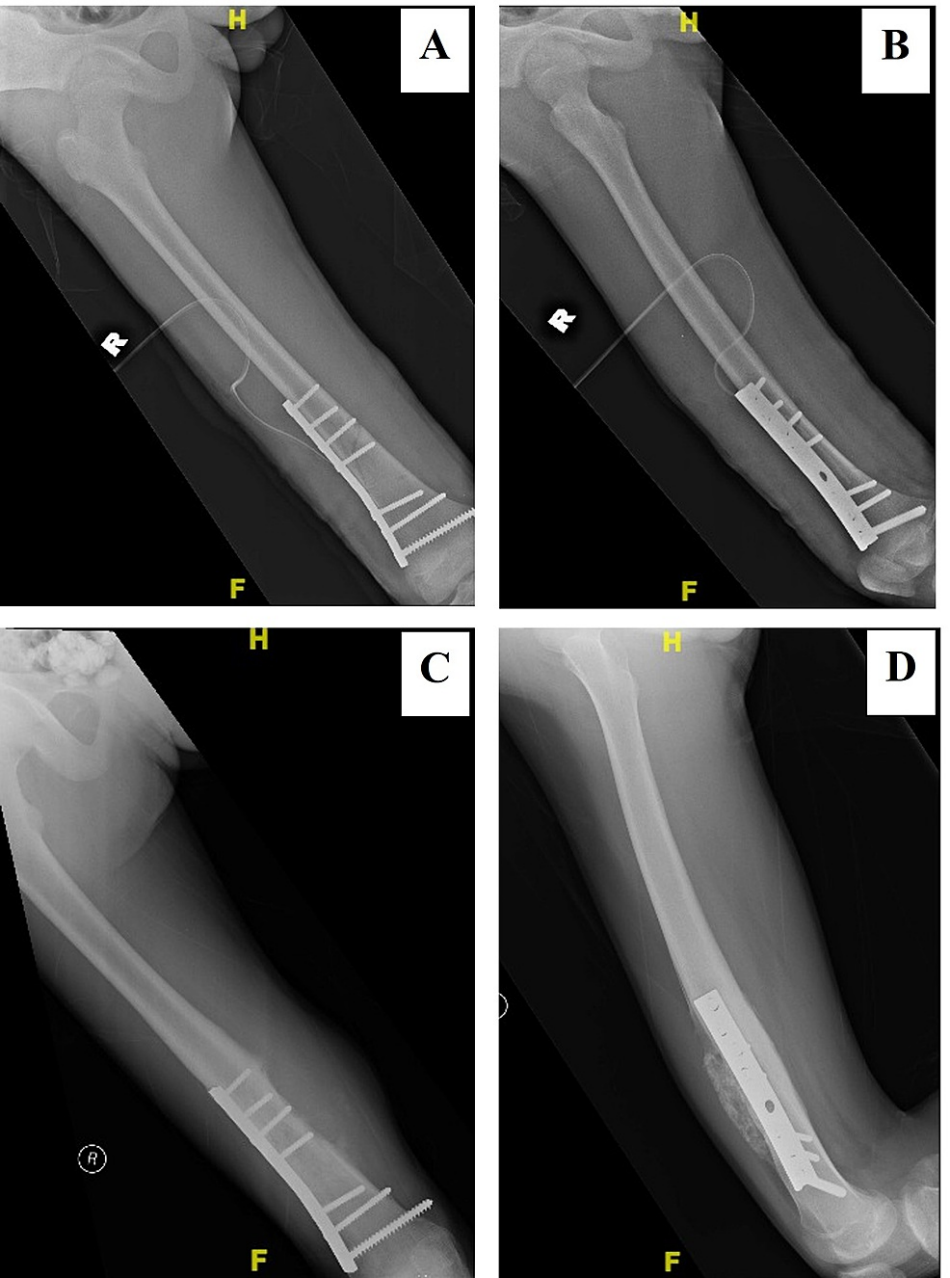
A distal third right femoral fracture stabilized with a narrow dynamic compression plate The initial plain radiographs did not show any evidence of malignancy (A, B). Three years after the surgery, periosteal reaction with the Codman triangle was seen at the medial aspect of the united fracture (C) with calcification of its surrounding soft tissue at the anterior aspect (D). H: head or cranial/ top part of the limb, R: right, F: foot or caudal/ lower part of the limb.

**Figure 6:**
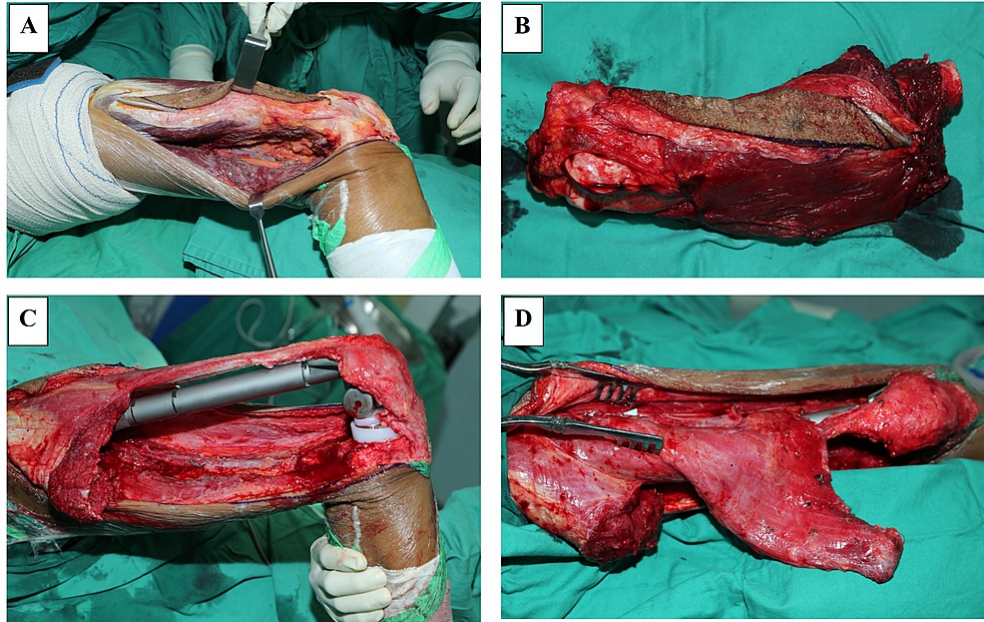
Intra-operative images of the second case during wide excision and reconstruction of the distal right femur Wide local excision of the distal right femur was performed (A, B) followed by limb reconstruction with a femur endoprosthesis and flap (C, D) due to the massive resection of soft tissue.

A 14-year-old boy sustained a closed distal third right femoral fracture due to a sports injury. He was treated with an open reduction and plating of the femur (Figures 5A, 5B). The fracture united well, and he regained full limb function. Three years later, he presented with painful swelling of his right thigh. Plain radiographs showed periosteal reaction with a Codman triangle at the united fracture site and calcification of the surrounding soft tissue (Figures 5C, 5D). Biopsy confirmed the diagnosis of osteosarcoma. Similar to Case 1, no metastasis was found during staging. He underwent three cycles of neoadjuvant chemotherapy according to the modified European Osteosarcoma Intergroup (EOI) Protocol and responded well. Wide local excision of the distal right femur was performed followed by limb reconstruction with a distal femur endoprosthesis and flap due to the massive resection of soft tissue (Figures 6A-6D). Postoperatively, he received another three cycles of adjuvant chemotherapy and local radiotherapy to which he responded well. He remained disease free during his last follow-up four years after the tumor surgery. He was able to ambulate without aid but with knee extension lag and knee flexion up to 90 degrees. Retrospective analysis of the initial trauma radiographs did not show any evidence of malignancy, which was three years before the clinical presentation (Figures 5A, 5B).

Case 3

**Figure 7:**
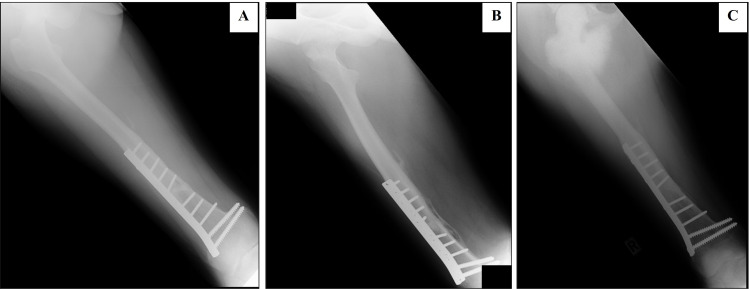
Plain radiographs of a closed fracture of the distal third right femur stabilized with a broad dynamic compression plate The fracture united with thick periosteal reaction and callus formation. No bony lesion was seen (A, B). Three years after the surgery, an osteoblastic lesion involving the metaphysis of the proximal ipsilateral was seen. There were extensive periosteal reaction and extra-osseous extension of the lesion medially. No obvious extension to the distal half of the femur. The femoral plate remained in situ (C).

**Figure 8:**
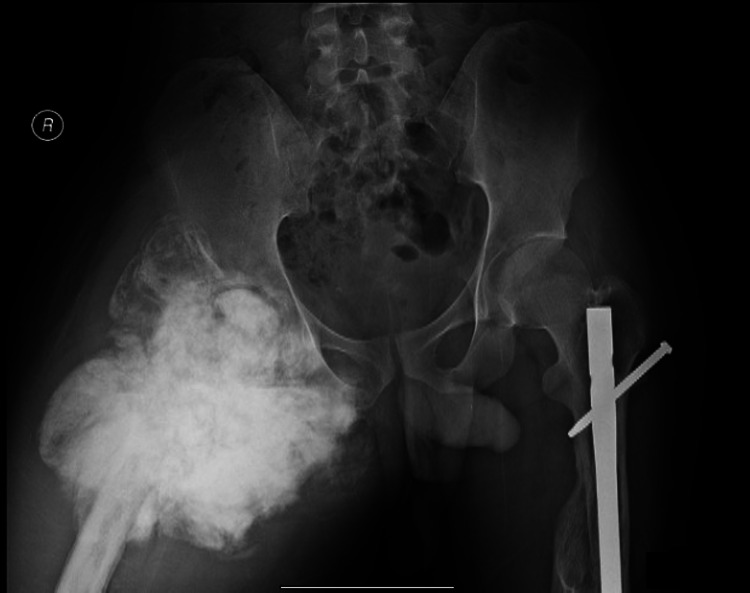
The plain radiograph one year before the patient succumbed to death There was an extensive involvement of the proximal right femur with no evidence of a similar lesion within the visualized contralateral femur. The left femoral nail remained in situ. R: right.

A 17-year-old boy had a severe head injury and multiple fractures following a motor vehicle accident. He was ventilated for three weeks and subsequently underwent multiple fracture fixations. The closed fracture of the distal third of his right femur was stabilized with a dynamic compression plate (Figures 7A, 7B). The fracture healed well, and he regained full limb function. Three years later, he presented with pain and swelling of the right hip. Plain radiographs revealed an osteoblastic lesion with soft tissue extension (Figure 7C). Biopsy confirmed the diagnosis of osteosarcoma. The initial staging did not find any evidence of metastasis. He was treated with three cycles of cisplatin and doxorubicin, but the tumor did not respond well to the chemotherapy (Figure 8) and subsequently metastasize to the lungs six months after the tumor was first detected. He succumbed to the disease a year later. The last radiograph before the genesis of osteosarcoma was one year following fracture fixation during a routine follow-up. There was no radiological evidence of bony lesion to suggest early malignancy. The disease behaved conventionally despite previous surgical procedures within the area, and the initial radiograph was not suspicious during the early stage.

## Discussion

Dorrestijn et al. reported an interesting case of osteosarcoma in the distal femur two years after an ipsilateral femur fracture [2]. Retrospectively, they concluded that it was a pathological fracture and complicated with non-union. The patient underwent multiple surgical procedures and subsequently manifested clinically and radiologically as osteosarcoma. Our third case highlighted the initial destructive process of osteosarcoma that occurred within the metaphyseal cancellous region, which was distant from the fracture site (Figure 3C). Based on the nature of the trauma and the injuries sustained, the femur had suffered a significant high-energy impact.

Osteosarcoma occurring at a previous fracture site in humans is very rare. Our literature search yielded only two reported cases in humans. Berry et al. reported a 12-year-old boy who developed osteosarcoma at a healed midshaft femoral fracture seven years after a multiple high-energy trauma [5]. Another case reported by Rao et al. described a 14-year-old boy who sustained a midshaft femoral fracture following a bicycle accident [6]. The fracture healed well after conservative treatment. However, osteosarcoma developed at the site of the united fracture after 10 years. In our second and third cases, we highlighted osteosarcoma that developed following high-energy trauma. These occurred without any prior clinical symptoms or signs of malignancy on the trauma plain radiographs. A retrospective cohort study of 163 osteosarcoma patients with an age range between six and 59 years treated at a single local oncology referral center in a five-year period reported that overall survival was highly related to the presence of pulmonary metastases and compliance to the administrated treatment. Significantly poorer survival was seen in patients who did not complete the treatment [7].

In cases of osteosarcoma, a fracture treated with an interlocking nail will result in gross contamination of the marrow and surrounding soft tissue [8]. The reaming procedure through the tumor will contaminate the entire femoral medullary cavity. The pressure generated from the reaming and fracture manipulation will also expand the hematoma and contaminate the entire thigh muscle compartment. However, there was no evidence of contamination in our first case (Figures 1D, 1E). The fracture united within a normal duration and healing was not hampered by the sarcoma. We postulate that in early disease, the local immunological response will contain the tumor against contamination. The symptoms will only appear when there is cortical destruction and soft tissue extension.

Our third case highlighted that osteosarcoma could occur in a perfectly normal bone away from the fracture and implant. It was documented that the proximal femur was normal during the initial serial evaluation of the fracture. Detailed cancellous and trabecular patterns also revealed no abnormalities. Subsequent radiographs demonstrated the presence of osteosarcoma that occurred three years later. This scenario raised a question for radiological screening to evaluate patients with a high risk of developing osteosarcomas such as in Li-Fraumeni syndrome and retinoblastoma. The sensitivity of radiological changes was not presented in early disease prior to clinical manifestation.

## Conclusions

Our three cases of osteosarcoma presentation following high-energy trauma are rare and unique. Initial trauma radiographs or clinical presentation without signs of malignancy does not exclude the risk of malignancy later in life. Young postoperative patients after high-energy trauma may require a regular and longer follow-up especially those with risk factors to enable early detection of any malignant changes and early intervention.

## References

[1] Goyal S, Roscoe J, Ryder WD, Gattamaneni HR, Eden TO (2004). Symptom interval in young people with bone cancer. Eur J Cancer.

[2] Dorrestijn O, Jutte PC (2011). Osteosarcoma in the distal femur two years after an ipsilateral femoral shaft fracture: a case report. J Med Case Rep.

[3] Widhe B, Widhe T (2000). Initial symptoms and clinical features in osteosarcoma and Ewing sarcoma. J Bone Joint Surg Am.

[4] Song WS, Jeon DG, Cho WH, Kong CB, Cho SH, Lee JW, Lee SY (2014). Plain radiologic findings and chronological changes of incipient phase osteosarcoma overlooked by primary physicians. Clin Orthop Surg.

[5] Berry MP, Jenkin RD, Fornasier VL, Rideout DF (1980). Osteosarcoma at the site of previous fracture. A case report. J Bone Joint Surg Am.

[6] Rao PT, Pradhan NK, Acharya S (1991). Osteosarcoma following a fractured shaft of femur. A case report. Int Orthop.

[7] Faisham WI, Mat Saad AZ, Alsaigh LN (2017). Prognostic factors and survival rate of osteosarcoma: a single-institution study. Asia Pac J Clin Oncol.

[8] Abudu A, Sferopoulos NK, Tillman RM, Carter SR, Grimer RJ (1996). The surgical treatment and outcome of pathological fractures in localised osteosarcoma. J Bone Joint Surg Br.

